# Nitrogen- and Sulfur-Codoped Strong Green Fluorescent Carbon Dots for the Highly Specific Quantification of Quercetin in Food Samples

**DOI:** 10.3390/ma16247686

**Published:** 2023-12-17

**Authors:** Kandasamy Sasikumar, Ramar Rajamanikandan, Heongkyu Ju

**Affiliations:** Department of Physics, Gachon University, Seongnam-si 13120, Gyeonggi-do, Republic of Korea; sasiphy2012@gmail.com (K.S.); chemistrmkd@gmail.com (R.R.)

**Keywords:** carbon dots, heteroatom doping, inner filter effect, p-phenylenediamine, quercetin, thioacetamide

## Abstract

Carbon dots (CDs) doped with heteroatoms have garnered significant interest due to their chemically modifiable luminescence properties. Herein, nitrogen- and sulfur-codoped carbon dots (NS-CDs) were successfully prepared using p-phenylenediamine and thioacetamide via a facile process. The as-developed NS-CDs had high photostability against photobleaching, good water dispersibility, and excitation-independent spectral emission properties due to the abundant amino and sulfur functional groups on their surface. The wine-red-colored NS-CDs exhibited strong green emission with a large Stokes shift of up to 125 nm upon the excitation wavelength of 375 nm, with a high quantum yield (QY) of 28%. The novel NS-CDs revealed excellent sensitivity for quercetin (QT) detection via the fluorescence quenching effect, with a low detection limit of 17.3 nM within the linear range of 0–29.7 μM. The fluorescence was quenched only when QT was brought near the NS-CDs. This QT-induced quenching occurred through the strong inner filter effect (IFE) and the complex bound state formed between the ground-state QT and excited-state NS-CDs. The quenching-based detection strategies also demonstrated good specificity for QT over various interferents (phenols, biomolecules, amino acids, metal ions, and flavonoids). Moreover, this approach could be effectively applied to the quantitative detection of QT (with good sensing recovery) in real food samples such as red wine and onion samples. The present work, consequently, suggests that NS-CDs may open the door to the sensitive and specific detection of QT in food samples in a cost-effective and straightforward manner.

## 1. Introduction

Flavonoids are secondary metabolites that belong to the polyphenolic compounds. Quercetin (3,3′,4′,5,7-pentahydroxyflavone; QT) is a member of the flavonol subclass in the flavonoid family and is naturally found in a wide range of vegetables, fruits, and beverages. It abundantly exists in capers, onions, broccoli, apples, cherries, and berries, as well as in red wine, cocoa, and tea [1]. QT has been the subject of interest owing to its in vitro antioxidant properties for scavenging free radicals, which are favorable for human health and disease prevention [2,3]. Moreover, the catechol ring and OH groups in its chemical structure qualify QT to partake in potent biological activities, including antiviral, antiallergic, anti-inflammatory, antimutagenic, anticarcinogenic, and cardioprotective activities [4,5]. However, high dietary consumption of QT may lead to headaches, renal failure, and poor glutathione S-transferase activity [6,7]. Therefore, the detection of QT is highly crucial for pharmaceutical chemistry, biochemistry, and clinical medicine.

Traditional methods available for the detection of QT, including spectrophotometry, electrochemical detection, capillary electrophoresis, and high-performance liquid chromatography (HPLC), are restricted in their application due to the limitations resulting from sophisticated instrumentation, operational difficulty, sluggish real-time responsivity, and poor sensitivity [8,9,10,11]. On the other hand, the fluorescence-based analytical technique has generated much attention for the quantitative detection of various organic and inorganic analytes because of its benefits over other approaches, including its rapid responsivity, superior sensitivity, high specificity, inherent simplicity, and low operating costs [12,13,14]. Fluorophores usually include metal nanoclusters, semiconductor quantum dots (QDs), and organic dyes [15,16,17]. Despite their high quantum yield and chemical stability, these materials are usually very toxic and complex to synthesize [18].

Zero-dimensional (0D) carbon-based nanomaterials, known as carbon quantum dots or carbon dots (CDs), have been gaining popularity as a novel class of sensing probes in fluorescence-based analytical techniques [19]. CDs, quasi-spherical nanomaterials with size confinement below 10 nm, were accidentally discovered in 2004 during the purification process of single-walled carbon nanotubes (CNTs) [20]. They have many advantages, such as tunable spectral luminescence, reduced overlap between excitation and emission spectra, good biocompatibility, low toxicity, high chemical stability, excellent photostability, good water-dispersibility, and the availability of abundant sources [21]. These special qualities lead them to be exploited in biomedicine, catalysis, optoelectronics, and sensing [22]. Still, most of the fabricated CDs have a lower quantum yield (QY) than conventional QDs, which limits their practical use [23,24].

The fluorescence characteristics of CDs arise from the quantum size effects, carbon-core states, conjugated π-domains, molecule states, and surface states [25,26]. It is highly expected that surface states are the main source of fluorescence—so-called fluorescence centers, created by the synergistic hybridization of carbon-core and associated chemical groups [27]. Very recently, heteroatom doping has become a more convenient strategy for engineering the surface states and electron distribution in CDs to greatly enhance their fluorescence properties [28]. The introduction of heteroatoms would create more surface states for electron trapping, which facilitates improved radiative recombination that results in a high QY, excitation-independent emission, and emission redshift [27,29]. Among the various heteroatoms for doping, nitrogen is a popular dopant because its atomic radius (0.075 nm) is comparable to that of carbon (0.077 nm). Similarly, the electronegativity of sulfur (2.58) is nearly equivalent to that of carbon (2.55), which enables easier electron transition [30,31]. Sulfur also significantly increases the density of graphitic nitrogen, which results in a redshift in the emission spectrum [32]. Hence, the codoping of nitrogen (N) and sulfur (S) atoms into CDs allows us to expect the synergistic effect of the two individual dopants.

**Scheme 1:**
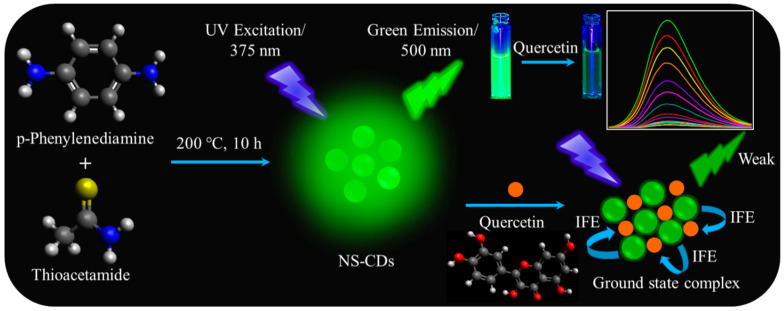
Synthesis of NS-CDs and detection of QT by fluorescence quenching.

We fabricated N- and S-codoped CDs (NS-CDs) using two simple precursors, i.e., p-phenylenediamine (p-PD) and thioacetamide, through a one-step solvothermal route with ethanol as a solvent. The p-PD has two –NH_2_ groups, while thioacetamide has C–NH_2_ and C=S groups. In contrast to previously reported CDs for QT sensing, the current NS-CDs were prepared under mild reaction conditions without any acids, complex molecules, or metal salts. The as-synthesized NS-CDs exhibited a large Stokes shift (~125 nm) with green emission for the excitation at 375 nm. However, the fluorescence was effectively quenched by QT through the inner filter effect (IFE), with a noticeable color change from green to a colorless solution. The quenching-based sensing strategy offered the lowest detection limit of 17.3 nM within the linear range of 0–29.7 μM, with good specificity against various interfering elements. The present work enabled the quantification of QT in food samples, which would be beneficial for maintaining good health and preventing chronic diseases. A diagram of the NS-CDs’ synthesis and QT detection is given in Scheme 1.

## 2. Materials and Methods

### 2.1. Chemicals

The p-PD, thioacetamide, hydroquinone (HQ), bovine serum albumin (BSA), resorcinol (Res), catechol (Cat), serotonin (Ser), dopamine (Dop), cysteine (Cys), and methionine (Met) were procured from Sigma Aldrich, St. Louis, MO, USA. Lysine (Lys), rutin trihydrate (Rut), and ethanol were supplied by Daejung Chemicals, Siheung, Republic of Korea. All of the reagents were used directly, without any purification. For all of the analyses, ultrapure water (18.2 MΩ-cm) collected from the Milli-Q system (Millipore, Burlington, VT, USA) was used.

### 2.2. Preparation of NS-CDs

Typically, 0.108 g of p-PD was weighed and dissolved in 5 mL of ethanol to prepare a 0.1 M solution of p-PD. With this solution, 0.15 M thioacetamide (0.112 g) dissolved in 5 mL of ethanol was gently mixed on a magnetic stirrer. The solution (net volume of 10 mL) was ultrasonicated for 5 min and then shifted to a 50 mL Teflon liner covered by a stainless steel vessel. The reactor was permitted to undergo a solvothermal reaction for 10 h inside an oven (Daihan Scientific, Wonju, Republic of Korea) at 180 °C temperature. After the reaction time was completed, the solution was spontaneously cooled to room temperature and centrifuged using a centrifuge (Combi-514R, Hanil Co., Ltd., Seoul, Republic of Korea) at 10,000 rpm for 10 min. Subsequently, the supernatant was lyophilized at a temperature of −80 °C using a freeze-dryer (FD8508, IlShinBioBase, Seoul, Republic of Korea) and stored at 4 °C for further use. The amount of NS-CDs recovered at the end of the preparation was 0.084 g.

### 2.3. Characterization of NS-CDs

Transmission electron microscopy (TEM) images were collected using a transmission electron microscope (Tecnai G2 F30, FEI, Hillsboro, Oregon, USA) at an operating voltage of 300 kV. The chemical compositions were examined using an X-ray photoelectron spectroscope with a monochromatic Al Kα source (VG MultiLab/2000, Thermo Scientific, Waltham, MA, USA). Fourier-transform infrared (FT-IR) spectra were measured using an FT-IR spectrometer (FT-IR/4600, JASCO, Tokyo, Japan). UV–vis absorption was studied on a spectrophotometer (V/770, JASCO, Tokyo, Japan). Photoluminescence spectra were recorded on a fluorescence spectrophotometer (FS/2, SCINCO, Seoul, Republic of Korea) at room temperature using a 1 cm path-length quartz cell. The slit width was set to 5 nm for both excitation and emission. The relative QY (Ф) was determined by measuring the emission intensity with a spectrofluorometer (Fluorolog3, Horiba, Tokyo, Japan). Fluorescence lifetime measurements were conducted by Fluorolog3 (Horiba, Tokyo, Japan) with a time-correlated single-photon counting instrument equipped with a 390 nm laser.

### 2.4. Quantum Yield (Ф) Measurement

The relative QY of NS-CDs, i.e., Ф_CD_, was determined by a standard method using rhodamine B as a reference (Ф_R_ = 31% in water), with the equation given by [33]:

(1) $${Ф}_{CD}={Ф}_{R}\frac{{I_{CD}A}_{R}}{{I_{R}A}_{CD}}{\left(\frac{n_{CD}}{n_{R}}\right)}^{2}$$

where I is the integrated emission intensity. The subscripts “CD” and “R” represent the NS-CDs and rhodamine B, respectively. A is the absorbance and n is the refractive index of the solvent. Both NS-CDs and rhodamine B were dissolved in water. Hence, the values of n_CD_ and n_R_ were taken as 1.33. The absorbance of fluorophore solutions was maintained below 0.05 to minimize the reabsorption of emitted light.

### 2.5. Fluorescence Sensing of QT

To determine the fluorescence response of NS-CDs towards QT, the NS-CDs solution (500 μL) was dispersed in phosphate buffer (10 mM, pH = 7). Then, the final volume of the solution was adjusted to 3 mL by adding water and used as a fluorescence probe. After 2 min of incubation time, the solution was excited at 375 nm, and the fluorescence intensity (F_0_) of the emission at 500 nm was recorded in the wavelength range of 470–700 nm. Successively, various quantities of quercetin (3.3–39.6 μL) were introduced into the NS-CDs solution, and the fluorescence intensities were recorded. F_0_ and F are the fluorescence intensities of the NS-CDs in the absence and presence of QT, respectively. To study the specificity of the NS-CDs towards QT, common potential interferents (e.g., phenols, biomolecules, amino acids, metal ions, and flavonoids) were introduced into the NS-CDs solution under the same experimental conditions. The measurements were repeated five times (n = 5).

### 2.6. Recovery Test in Food Samples

Commercial red wine and onion were bought from a local supermarket (Emart, Seongnam, South Korea) for the purpose of real sample analysis of the NS-CDs fluorescence probe. The red wine sample was used directly as purchased. To prepare the onion sample, onion paste was made, centrifuged at 10,000 rpm for 10 min, filtered through a 0.45 μm filter membrane to remove the impurities, and then stored at 4 °C for analysis. The recovery study was performed by adding different concentrations of standard QT (0, 5.0, 10.0, 15.0, and 30.0 μM) to the red wine and onion samples in an aqueous medium. The pH value of all sample solutions was regulated to 7. The recovery (%) was estimated from the following equation [34]:

(2) $$Recovery\left(\%\right)=\frac{Amount\;of\;QT\;detected}{Amount\;of\;QT\;spiked}\times 100$$

## 3. Results and Discussion

### 3.1. Analysis of Morphology and Chemical Compositions

**Figure 1:**
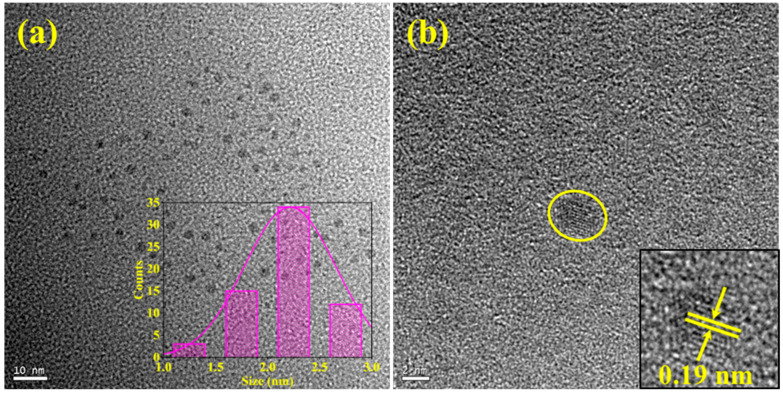
(**a**) TEM photograph of NS-CDs. Inset: histogram of particle size distribution. (**b**) HR-TEM image of a single NS-CD. Inset: the corresponding lattice fringes.

The morphology and size distribution of the NS-CDs were examined using TEM and HR-TEM. Figure 1a displays a TEM image of NS-CDs that are spherical with uniform dispersibility. The particles are distributed between 1.1 nm and 3.7 nm, with an average size of 2.2 ± 0.41 nm (inset of Figure 1a). The average particle size and standard deviation were calculated from the histogram plotted by measuring the diameter of 65 particles using ImageJ 1.41 software. As shown in Figure 1b, the synthesized NS-CDs exhibited good crystallinity, with an interplanar spacing of 0.19 nm (inset of Figure 1b), which coincides with the (102) diffraction facets of graphitic carbon [35].

The functional groups on the surface of the NS-CDs were studied through FT-IR spectroscopy. In Figure S1, the peaks at 3346 cm^−1^ and 2972 cm^−1^ can be linked to the stretching vibrations of the N–H and C–H groups, respectively. The characteristic peaks centered at 1743 cm^−1^ and 1653 cm^−1^ correspond to the stretching vibrations of the C=C group. The peak located at 1382 cm^−1^ can be ascribed to the deformation vibrations of the O–H group. The peaks at 1084 cm^−1^ and 1045 cm^−1^ are assigned to the stretching vibrations of the C=S/C–O and C–N groups, respectively. The peaks at 833 cm^−1^ and 630 cm^−1^ are associated with the deformation vibrations and rocking vibrations of the C–H group, respectively [36,37,38]. The FT-IR spectral results confirm the existence of amino- and sulfur-containing groups on the NS-CDs’ surface.

**Figure 2:**
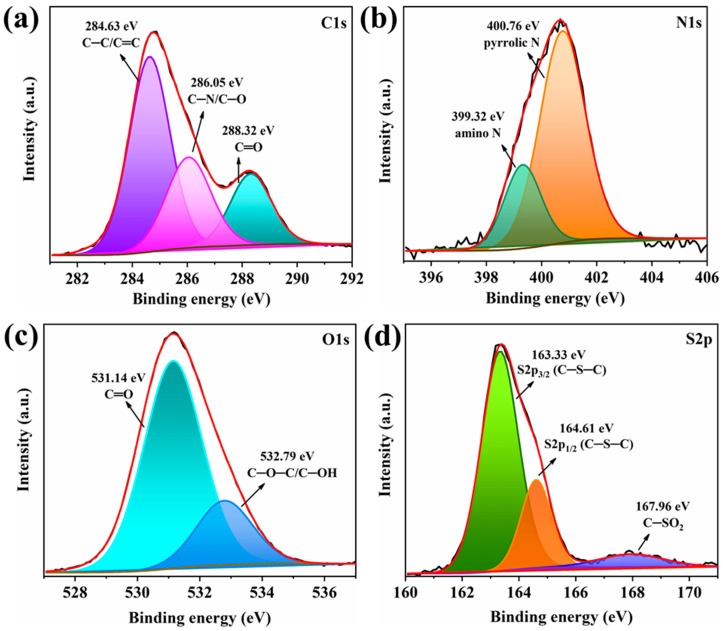
High-resolution XPS spectra of (**a**) C1s, (**b**) N1s, (**c**) O1s, and (**d**) S2p of the NS-CDs.

Moreover, the surface chemical states and elemental compositions of the NS-CDs were revealed by XPS analysis. In Figure S2, the XPS survey scan discloses four peaks around 163.93 eV, 285.16 eV, 400.66 eV, and 531.45 eV, corresponding to S2p, C1s, N1s, and O1s, respectively. This indicates that the N and S atoms were successfully doped into the CDs. Particularly, the deconvoluted C1s spectrum in Figure 2a can be resolved into three different peaks at 284.63 eV, 286.05 eV, and 288.32 eV, which can be attributed to C–C/C=C, C–N/C–O, and C=O, respectively [39]. In the high-resolution N1s spectrum (Figure 2b), the two peaks located at 399.32 eV and 400.76 eV are due to the amino-N and pyrrolic-N groups, respectively [40]. The high-resolution O1s spectrum (Figure 2c) exhibits two peaks at 531.14 eV and 532.79 eV, which are assigned to the C=O and C–O–C/C–OH groups, respectively. The high-resolution S2p spectrum (Figure 2d) consists of two characteristic peaks at 163.33 eV and 164.61 eV, arising from the 2p_3/2_ and 2p_1/2_ positions in the C–S–C covalent bonds due to the spin–orbit coupling, respectively. Another peak located at 167.96 eV can be attributed to the presence of –C–SO*_x_*− (*x* = 2) species [39,41]. The XPS analysis results are in good agreement with the FT-IR measurements, verifying that the synthesized NS-CDs have rich nitrogen and sulfur groups to qualify NS-CDs with good luminescence properties.

### 3.2. Optical Properties

**Figure 3:**
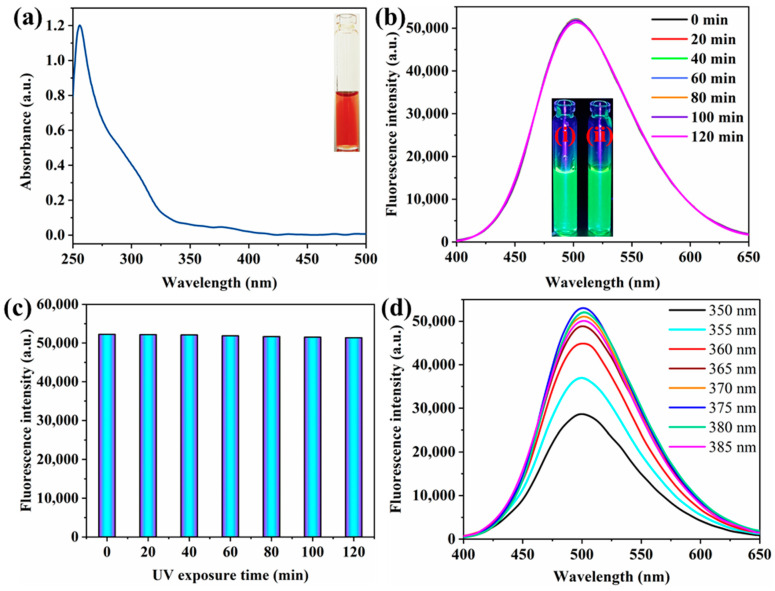
(**a**) UV–vis spectrum of NS-CDs. Inset: photograph of NS-CDs in visible light. (**b**) Fluorescence emission spectra of NS-CDs at different times of UV light exposure (λ = 365 nm). Inset: photographs of NS-CDs under UV light for (i) 0 min and (ii) 120 min. (**c**) Bar chart showing the photostability of NS-CDs in different time intervals under UV light. (**d**) Changes in the fluorescence emission intensity of NS-CDs at different excitation wavelengths ranging from 350 nm to 385 nm.

The optical properties of the NS-CDs were investigated using UV–vis absorption and fluorescence spectra. In Figure 3a, a wide absorption region around 300 nm arises from the π–π* transitions of the aromatic sp^2^ domains [42]. Another absorption peak at 377 nm can be ascribed to the trapping of excited-state energy of the surface states contributed by the functional groups connected to the surface of the NS-CDs [43]. This peak might be consistent with the optimal excitation peak for NS-CDs. As shown in the inset of Figure 3a, the aqueous solution of NS-CDs was a wine-red color in visible light and displayed a strong green fluorescence under UV illumination (λ = 365 nm), illustrating the excellent luminescence properties of NS-CDs with good dispersibility. The photostability of the NS-CDs was verified at different time intervals (0, 20, 40, 60, 80, 100, and 120 min) by illuminating the NS-CDs with UV light continuously for up to 120 min. As displayed in Figure 3b, the fluorescence intensities were not significantly different before and after the UV illumination. This is further detailed by the bar chart in Figure 3c, showing that the NS-CDs have excellent photostability against photobleaching.

Figure 3d displays the fluorescence emission spectra of the NS-CDs for different excitation wavelengths ranging between 350 nm and 385 nm. The position of the emission peak was not shifted with respect to the excitation wavelength, and the strongest emission peak appeared at 500 nm for the excitation at 375 nm, which is well consistent with the UV–vis absorption spectrum. This excitation-independent emission behavior arises from the homogeneous surface states’ emissive trap sites, and it could also be useful in condensing the interfering effect of autofluorescence during the analyte detection [44]. Hence, the excitation and emission wavelengths were set at 375 nm and 500 nm, respectively, to record the fluorescence intensity. The Stokes shift was about 125 nm, indicating the potential of NS-CDs for analytical application. Moreover, the QY of the NS-CDs was determined under 375 nm excitation with reference to rhodamine B, and a high QY of 28% was achieved. This is because the N–S-codoping increases the degree of conjugated π-domains or makes it easier for electrons to be trapped by the newly developed surface states, thereby promoting a high yield of radiative recombination [29].

### 3.3. Optimized Conditions

The parameters that could affect the performance of the NS-CDs fluorescence probe in the detection of QT were optimized. First, to ascertain the kinetic response of the fluorescence probe, the incubation time was monitored for 30 min. The fluorescence response of the NS-CDs was checked every 2 min following the addition of QT (10 μM). As shown in Figure S3, QT could completely react with the NS-CDs within 2 min, which resulted in a sharp decrease in fluorescence intensity. Following this time, the fluorescence intensity was almost stable, suggesting that 2 min would be the ideal incubation time for the sensing studies.

Next, the effect of pH (3.0–9.0) on the fluorescence intensity of the NS-CDs was investigated. As displayed in Figure S4, it could be observed that the fluorescence intensity gradually increased in the pH range of 3.0–7.0. Afterward, the fluorescence intensity did not remarkably change up to the pH value of 9.0. As the highest fluorescence intensity of the NS-CDs was attained at pH ~7.0, this value was selected as the optimal pH for QT detection.

### 3.4. Sensitive Detection of QT

**Figure 4:**
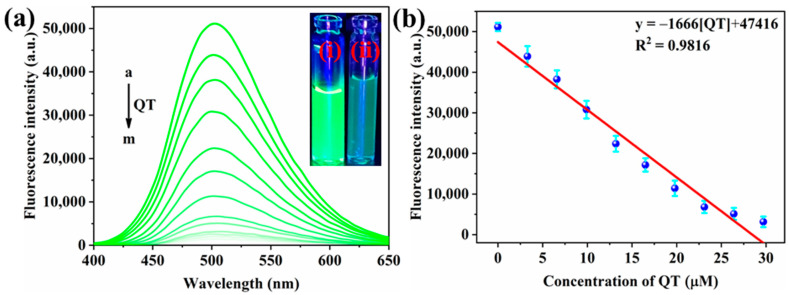
(**a**) Quenching of the fluorescence intensity of NS-CDs with the addition of QT increasing in steps of 3.3 μM (a→m: 0–39.6 μM) (λ_ex_ = 375 nm; λ_em_ = 500 nm). Inset: photographs of NS-CDs (i) without QT and (ii) with QT (39.6 μM). (**b**) Plot of fluorescence intensity vs. QT concentration within the linear range of 0–29.7 μM.

As the developed NS-CDs probe exhibited excellent fluorescence properties, the performance of the fluorescence probe in the quantitative detection of QT was examined by carrying out titration experiments under optimal conditions. For the excitation at 375 nm, the fluorescence intensity of the NS-CDs was exactly 500 nm. As shown in Figure 4a, the fluorescence intensity was sensitive to QT and was systematically reduced with the increase in the QT concentration (0–39.6 μM), signifying a typical concentration-dependent behavior of the probe. Upon increasing the concentration to 39.6 μM, the fluorescence of the NS-CDs was almost quenched by 97%, signifying the competence of the proposed fluorescence probe. As shown in Figure 4b, the fluorescence intensity of the NS-CDs has a good linear relationship with the concentration of QT in the range between 0 and 29.7 μM. This linear portion of the plot can be fitted into the equation F_0_/F = −1666[QT] + 47,416, R^2^ = 0.9816, where *F*_0_ and F represent the fluorescence intensities of the NS-CDs without QT and with QT, respectively, and [QT] is the quencher’s concentration. The LOD was determined from the equation LOD = 3σ/m, where σ is the standard deviation obtained from the blank measurement (n = 5) and m is the slope acquired from the linear plot [45]. The value of the LOD was 17.3 nM. In terms of the linear detection range and LOD, a comparison of the current fluorescence probe with previously reported probes is given in Table 1. Obviously, the LOD of the present approach is significantly lower than in other reported works, which reveals that NS-CDs can perform well in practical fluorescence-based analytical applications.

### 3.5. Fluorescence Quenching Mechanism

**Figure 5:**
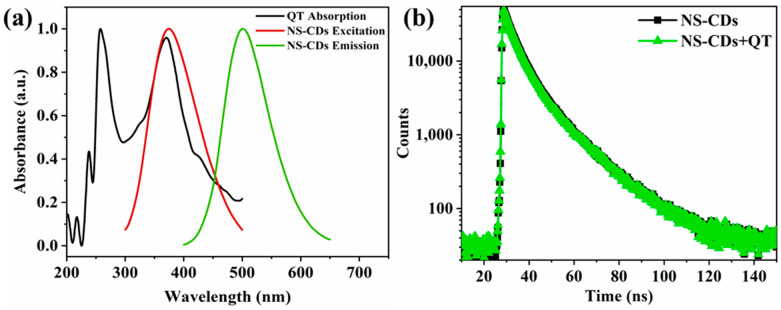
(**a**) Spectral overlap between absorption of QT and excitation of NS-CDs. (**b**) Fluorescence lifetime decay of NS-CDs without QT and with QT (40 μM).

Of the various types of quenching mechanisms, Förster resonance energy transfer (FRET) ensues when the emission spectrum of the fluorophore overlaps the absorption spectrum of the quencher molecule, as well as if the distance between them is less than 10 nm [53]. In the case of the IFE, a spectral overlap occurs between the excitation and/or emission spectrum of the fluorophore and the absorption spectrum of the quencher molecule [54]. Also, in the presence of a quencher, the fluorescence lifetime of the fluorophore decreases in the FRET process, while it remains constant in the IFE process [55]. So, in order to reveal the quenching mechanism, the spectra of QT and NS-CDs were studied first. In Figure 5a, the absorption band of QT at 370 nm overlaps with the excitation band of the NS-CDs at 375 nm, suggesting that the QT-induced fluorescence quenching effect may be due to the presence of an IFE or FRET. To elucidate this further, the fluorescence lifetimes of the NS-CDs without QT (τ_0_) and with QT (τ_1_) were measured. The data were fitted using a triple exponential model and are shown in Figure 5b. The values were τ_0_ = 8.73 ns and τ_1_ = 8.54 ns for bare the NS-CDs and QT-added NS-CDs, respectively. As the lifetime values were almost the same, the possibility of the FRET mechanism could be ruled out. So, the IFE could be the main mechanism for the fluorescence quenching of NS-CDs.

Moreover, the quenching action of QT on the fluorescence response of NS-CDs can be classified into static and dynamic quenching, which can be evaluated by following the standard Stern–Volmer equation [56,57]:

F_0_/F = 1 + k_q_τ_0_[QT] = 1 + K_SV_[QT] (3)

where F_0_ and F are the fluorescence intensities of the fluorophore without and with the quencher, respectively, k_q_ is the bimolecular quenching constant, τ_0_ is the lifetime of the fluorophore without the quencher, [QT] is the quencher’s concentration, and K_SV_ is the Stern–Volmer constant. Static quenching is usually caused by means of the non-fluorescent ground-state complex formation between the fluorophore and the quencher molecule. In contrast, dynamic quenching occurs due to the collisions between the above molecular systems. It is commonly known that quenching action can be either static or dynamic if the Stern–Volmer plot displays a linear relationship; the coexistence of both static and dynamic quenching is evidenced by a nonlinear upward curve in the plot [58,59]. As shown in Figure S5, the Stern–Volmer plot establishes good linearity in the concentration range of 3.3–16.5 μM, with a regression coefficient (R^2^) of 0.9799. From the slope of the linear plot, K_SV_ was calculated to be 1.387 × 10^5^ M^−1^. Substituting the values of K_SV_ and τ_0_ in Equation (3), the quenching constant (k_q_) was determined to be 1.588 × 10^12^ M^−1^s^−1^. The obtained k_q_ value was much greater than the dynamic quenching constant (1.0 × 10^10^ M^−1^s^−1^), representing the static quenching process by the ground-state complex (non-fluorescent NS-CDs-QT complex) formation. This can be verified based on the theory of “hard and soft acids and bases (HSAB)”. According to this model, the high polarizable donor atoms (i.e., N, S heteroatoms) on the NS-CDs’ surface belong to the soft bases. Due to the hydroxyl groups attached to the aromatic rings in phenol, QT belongs to the soft acids. Consequently, an NS-CDs-QT complex might have formed as a result of the electrostatic interaction between the NS-CDs and QT, which may restrict the transfer of non-radiative electrons and cause the fluorescence intensity to decrease [49,60]. Overall, the NS-CDs showed good sensitivity and high specificity for QT detection due to the occurrence of a strong IFE and a remarkable static quenching process.

### 3.6. Specificity of QT Detection

**Figure 6:**
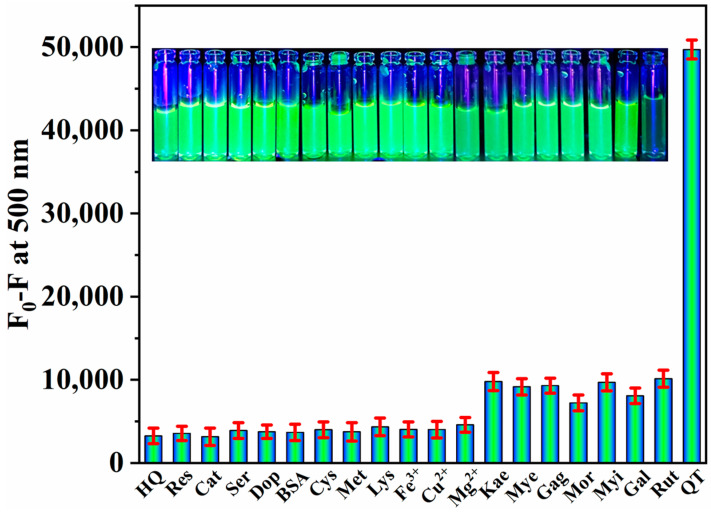
Specificity of NS-CDs to QT over potential interferents.

The specificity of the NS-CDs fluorescence probe for QT was appraised in ultrapure water based on the fluorescence change upon excitation at 375 nm. Various chemical compounds, such as phenols, biomolecules, amino acids, metal ions, and flavonoids, acted as potential interferents against QT. Hydroquinone (HQ), resorcinol (Res), catechol (Cat), serotonin (Ser), dopamine (Dop), bovine serum albumin (BSA), cysteine (Cys), methionine (Met), lysine (Lys), Fe^3+^, Cu^2+^, Mg^2+^, kaempferol (Kae), myricetin (Mye), galangin (Gag), morin (Mor), myricitrin (Myi), gallic acid (Gal), and rutin (Rut) were taken fivefold with NS-CDs, and the corresponding fluorescence intensity was recorded. As displayed in Figure 6, QT quenched the fluorescence intensity of NS-CDs effectively compared to the same concentration of other interferents. The poor quenching efficiency of some interferents for the fluorescence response of NS-CDs might be due to the minimal overlap between the excitation or fluorescence spectrum of NS-CDs and the absorption spectrum of the interferents, leading to a weak IFE.

### 3.7. Detection of QT in Food Samples

To determine the competence of the NS-CDs fluorescence probe, different QT concentrations (0, 5.0, 10.0, 15.0, and 30.0 μM) were added to red wine and onion samples, and the assessment was carried out by the standard addition method. After spiking the QT in each sample, the recovery was performed by measuring the fluorescence spectrum. The obtained results are listed in Table 2 with the relative standard deviation (RSD), indicating that the NS-CDs probe possesses good accuracy and high specificity to QT in food samples. Moreover, the recovery (93.87–102.27%) and RSD (1.08–2.86%) values are within the acceptable ranges. These results clearly suggest that the proposed NS-CD-based fluorescence probe offers significant value for QT detection in food samples.

## 4. Conclusions

Highly green fluorescent NS-CDs were prepared through a solvothermal method, using p-PD and thioacetamide as the precursors. The NS-CDs showed emission at 500 nm under the excitation at 375 nm. The excitation-independent emission and superior QY (28%) of the NS-CDs were achieved due to the uniform distribution of surface states by means of the amino and sulfur groups attached to the NS-CDs’ surface. Fluorescence quenching of the NS-CDs, caused by both a QT-induced IFE and ground-state complex formation, was used for the quantitative detection of QT. The LOD of 17.3 nM was achieved, reflecting very high sensitivity together with remarkable specificity to QT against various potential interferents. The NS-CD-based detection strategy also demonstrated the successful analysis of QT in red wine and onion samples, with good reproducibility in the range of 93.87–102.27%. These findings suggest that the detection strategy demonstrated can serve to quantify QT in food samples.

## Figures and Tables

**Table 1:** Comparison of NS-CDs with various fluorescence probes for QT detection.

Fluorescence Probe	Chemical Precursors	Linear Range (μM)	LOD (nM)	Ref.
Red-emissive CDs	Neutral red + catechol	0.66–111.33	190.0	[46]
Boron-functionalized CDs	Ethylenediamine + 4-boronobenzoyl chloride	0–28.5	28.8	[47]
N,Cu-codoped CDs	Ethylenediaminetetraacetic acid + CuCl_2_	0–100.0	75.4	[48]
N,S-codoped graphene quantum dots	Citric acid + cysteamine hydrochloride	0.17–33.09	56.25	[49]
Sulfur-doped graphene quantum dots	Citric acid + 3-mercaptopropionic acid	0–50.0	19.85	[50]
Organosilane-functionalized CDs (SiCDs)	Citric acid + N-(b-aminoethyl)-g-aminopropyl methyldimethoxy silane	0–40.0	79.0	[51]
Carbon nanodots	Glucose + ethylenediamine + nitric acid	1.0–47.0	172.4	[52]
**N,S-codoped CDs**	**p-Phenylenediamine + thioacetamide**	**0–29.7**	**17.3**	**This work**

**Table 2:** Recovery results of food samples spiked with QT at different standard levels.

Samples	Amount of QT Spiked (μM)	Amount of QT Detected (μM)	Recovery (%)	RSD * (%) (n = 5)
Red wine	0	4.38 ± 0.05	---	1.08
	5.0	9.24 ± 0.23	97.2	2.53
	10.0	13.97 ± 0.20	95.9	1.42
	15.0	18.46 ± 0.50	93.87	2.72
	30.0	33.91 ± 0.46	98.43	1.35
Onion	0	1.50 ± 0.04	---	2.56
	5.0	6.22 ± 0.12	94.4	1.91
	10.0	11.13 ± 0.32	96.3	2.86
	15.0	16.84 ± 0.19	102.27	1.13
	30.0	30.59 ± 0.81	96.97	2.64

* RSD: relative standard deviation = (standard deviation/average) × 100.

## Data Availability

Data are contained within the article and Supplementary Materials.

## Supplementary Materials

The following supporting information can be downloaded at: https://www.mdpi.com/article/10.3390/ma16247686/s1. Figure S1: FT-IR spectra of NS-CDs. Figure S2. XPS survey scan of NS-CDs. Figure S3. Effect of time on the fluorescence intensity of NS-CDs in the presence of QT (10 μM) at room temperature. Figure S4. pH-induced change in fluorescence intensity of NS-CDs at room temperature. Figure S5. Stern–Volmer plot of the quenching of fluorescence of NS-CDs by QT in the concentration range of 3.3–16.5 μM.
