# Supplementary material for: Nitrogen- and Sulfur-Codoped Strong Green Fluorescent Carbon Dots for the Highly Specific Quantification of Quercetin in Food Samples
Source: Materials (Basel). 2023 Dec 17;16(24):7686. doi: 10.3390/ma16247686 (PMC10744681; doi:10.3390/ma16247686)
Supplement: Supplementary file 1 [file materials-16-07686-s001.zip › materials-2765827-supplementary.pdf]

Supporting Information

S.No.	Captions	Page No.
**Figure S1.**	FT-IR spectra of NS-CDs.	S1
**Figure S2.**	XPS survey scan of NS-CDs.	S2
**Figure S3.**	Effect of time on the fluorescence intensity of NS-CDs in the presence of QT (10 µM) at room temperature.	S2
**Figure S4.**	pH-induced change in fluorescence intensity of NS-CDs at room temperature.	S3
**Figure S5.**	Stern–Volmer plot of the quenching of fluorescence of NS-CDs by QT in the concentration range of 3.3-16.5 µM.	S3

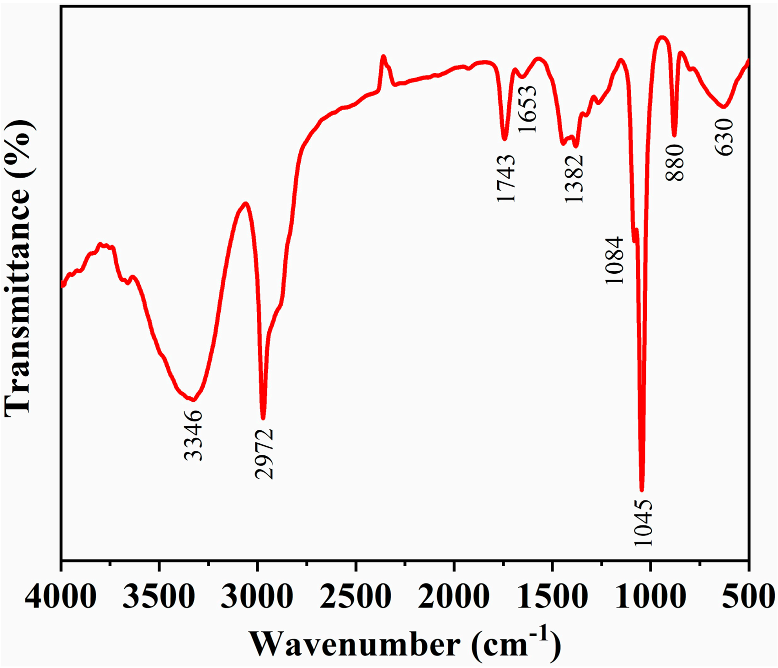

Figure S1. FT-IR spectra of NS-CDs.

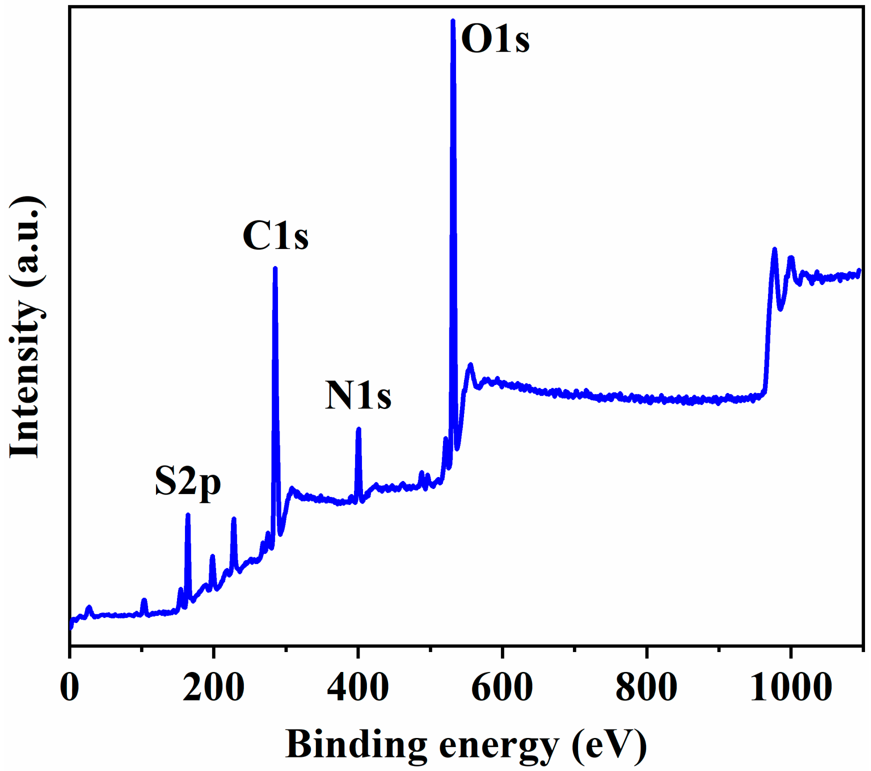

Figure S2. XPS survey scan of NS-CDs.

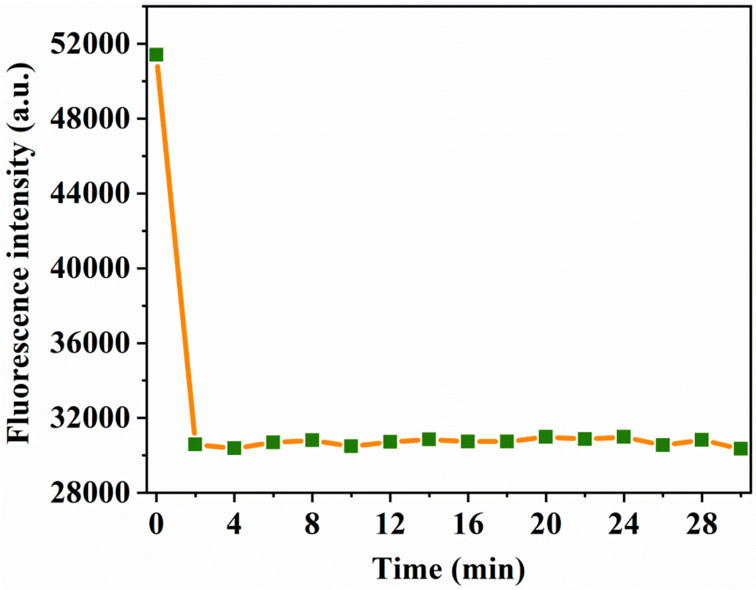

Figure S3. Effect of time on the fluorescence intensity of NS-CDs in the presence of QT (10 μM) at room temperature.

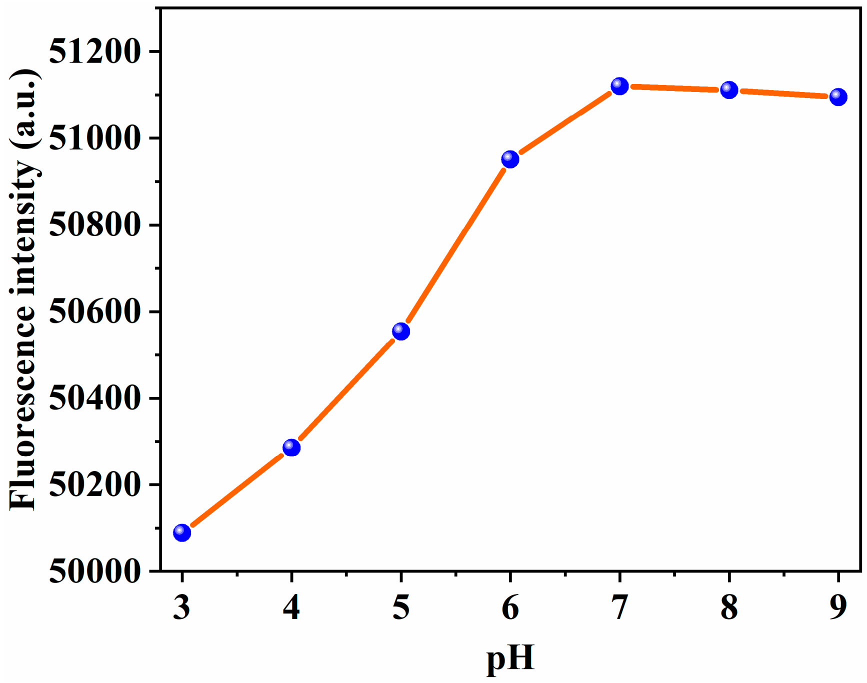

Figure S4. pH-induced change in fluorescence intensity of NS-CDs at room temperature.

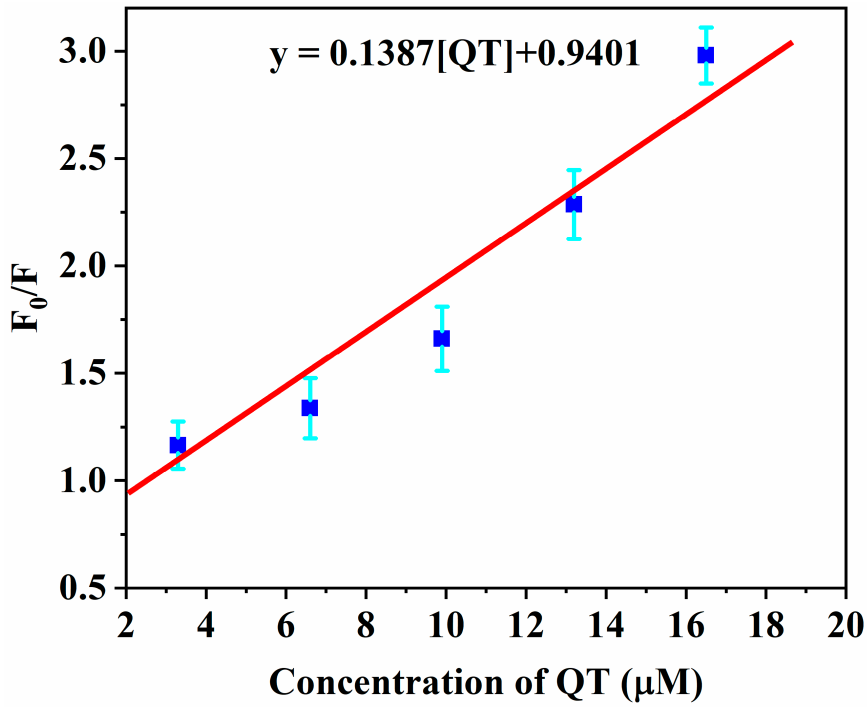

Figure S5. Stern–Volmer plot of the quenching of fluorescence of NS-CDs by QT in the concentration range of 3.3-16.5 μM.
